# A population-based study to assess two convolutional neural networks for dental age estimation

**DOI:** 10.1186/s12903-023-02817-2

**Published:** 2023-02-17

**Authors:** Jian Wang, Jiawei Dou, Jiaxuan Han, Guoqiang Li, Jiang Tao

**Affiliations:** 1 Department of General Dentistry, Shanghai Ninth People’s Hospital, Shanghai Jiao Tong University School of Medicine, College of Stomatology, Shanghai Jiao Tong University, Shanghai, 200011 China; 2 School of Software, Shanghai Jiao Tong University, Shanghai, 200240 China; 3 National Center for Stomatology, National Clinical Research Center for Oral Diseases, Shanghai Key Laboratory of Stomatology, Shanghai Research Institute of Stomatology, Shanghai, 200011 China

**Keywords:** Dental age estimation, Chinese population, Tooth development, Convolutional neural network, Orthopantomogram

## Abstract

**Background:**

Dental age (DA) estimation using two convolutional neural networks (CNNs), VGG16 and ResNet101, remains unexplored. In this study, we aimed to investigate the possibility of using artificial intelligence-based methods in an eastern Chinese population.

**Methods:**

A total of 9586 orthopantomograms (OPGs) (4054 boys and 5532 girls) of the Chinese Han population aged from 6 to 20 years were collected. DAs were automatically calculated using the two CNN model strategies. Accuracy, recall, precision, and F1 score of the models were used to evaluate VGG16 and ResNet101 for age estimation. An age threshold was also employed to evaluate the two CNN models.

**Results:**

The VGG16 network outperformed the ResNet101 network in terms of prediction performance. However, the model effect of VGG16 was less favorable than that in other age ranges in the 15–17 age group. The VGG16 network model prediction results for the younger age groups were acceptable. In the 6-to 8-year-old group, the accuracy of the VGG16 model can reach up to 93.63%, which was higher than the 88.73% accuracy of the ResNet101 network. The age threshold also implies that VGG16 has a smaller age-difference error.

**Conclusions:**

This study demonstrated that VGG16 performed better when dealing with DA estimation via OPGs than the ResNet101 network on a wholescale. CNNs such as VGG16 hold great promise for future use in clinical practice and forensic sciences.

**Supplementary Information:**

The online version contains supplementary material available at 10.1186/s12903-023-02817-2.

## Introduction

Chronological age reflects one’s typical biological characteristics and plays a vital role in a wide range of scenarios, such as distributing social welfare, selecting athletes in competitive sports, enrolling teenagers in schools, evaluating the ages of corpses, handling international refugees, adopting undocumented children of uncertain ages, and judging juvenile delinquencies. Therefore, the identification of an accurate and reliable method for estimating age is crucial [1–3].

Currently, there are three primary categories for estimating chronological age: laboratory-guided molecular biology studies, dental indicators, and bone markers [1]. Dental indicators have several advantages over the other two methods. Numerous studies have found that teeth have a strong link with maturity and are a reliable indicator for determining chronological age [2, 4–7]. Second, teeth are considered a dependable biological reflection of aging because of their durability and stability [8]. When using radiological dental age (DA) methods, DA estimation is also regarded as a noninvasive means of determining an individual’s age. The American Society of Forensic Odontology and the Study Group on Forensic Age Diagnostics recommend radiological DA estimation methods to assist in inferring chronological age [9, 10].

In 1973, Demirjian et al. observed a series of continuous DA development stages and divided them into eight phases, which started from the initial presence of a crypt (stage A) to the closure of the terminal tooth apices (stage H), based on orthopantomograms (OPGs) from a French-Canadian population [2]. Inspired by Demirjian and other pioneers in the scientific community, various methods have been developed, such as Willems method [4], Cameriere’s method [11], and London atlas [12]. Although different DA assessment methods have been performed reasonably well worldwide, few have been reported in China. Numerous studies have concluded that over- or underestimation of chronological age occurs, and an appropriate modification should be performed [1, 10–14]. To test the applicability of the Demirjian and Willems methods, we conducted two observational studies in Qingdao and Shanghai separately based on the local Chinese Han population aged 11 to 18 years, and similar results of underestimation of actual age were obtained using different data [13]. To determine the inner influential factors contributing to age estimation methods, another previous systemic and meta-analysis study selected the Willems method and explored its universality and accuracy at the global level [3]. Again, ethnicity-specific datasets have restricted regional applications. Linear and nonlinear models have been attempted [14, 15]; however, the accuracy of the two classic DA estimation methods is far from satisfactory.

In operating Demirjian DA assessment tasks, we found that the subjectivity and reproducibility of the technique measurement bias are their main limitations, despite the application of inter-/intra-observer agreement. Decoding the necessary parameters from an OPG also requires a certain amount of time. A report revealed that the mean time of estimating chronological age via an OPG was approximately 10 min [16]. When faced with large sample sizes, DA evaluation and manual performance are time-consuming and labor-intensive. If an optimal approach advancement can be achieved, it will be a huge leap for DA estimation.

In recent years, convolutional neural networks (CNN), a deep-learning technology, have been applied throughout the entire spectrum of pattern cognition, including picture recognition, target detection, and image classification [17–20]. CNN’s excellent pipelines may be demonstrated in mitotic pattern identification, chest computed tomography interstitial lung disease diagnosis, and fundus photo vascular segmentation. It consists of three main units: a convolutional layer, a pooling layer, and a fully linked layer. In the field of DA estimation, the technological advantage of CNN from the AI arsenal could provide hidden age information. Using data from Shanghai juveniles, we found that a machine-learning model based on multi-layer perceptron artificial neural networks (MLP-ANN) could considerably reduce the disparities between DA and chronological age [14]. Kim et al. also employed a CNN-based learning technique to visualize the four first molars in the mouth and retrieve anatomical data such as the pulp and alveolar bone [21]. This AI-based age group estimation model is reasonably valid, with an accuracy of 89.05%–90.27%. Furthermore, Merdietio Boedi R et al. performed an automated module cutting on the third molar of the left mandible [17] and Vila-Blanco et al. developed a model for predicting chronological age related to CNN [18]. Both studies exceeded expectations by shedding more information on the topic of age estimation. Despite limited experience with DA estimates, studies using the CNN method remain unclear. Is it possible for sophisticated CNN to improve traditional estimation? Therefore, the objectives of the present study were to investigate two novel CNN models (VGG and ResNet) based on a large-scale sample of OPGs (9586) and to test their validity in a teenagers’ DA estimation without the annotation of traditional mandibular left permanent teeth.

## Materials and methods

### Data acquisition and inclusion criteria

A total of 9586 OPGs comprised of 4054 males and 5532 females aged 6–20 years were included. The study group was separated into 15 age groups at a 1-year interval. Data were obtained from the database of the Department of Radiology, Shanghai Ninth People’s Hospital, Shanghai Jiao Tong University School of Medicine, from 2016 to 2020. All methods were performed according to the relevant guidelines and regulations. Informed consent was obtained from all participants and/or their legal guardians (s) for participation in the study.

The inclusion criteria were as follows: (1) no missing teeth in the left mandible (except the third molar), (2) normal tooth development and dental condition without serious jaw deformities or malocclusions, (3) clear and sufficient quality radiographs, and (4) eastern Chinese origin. Detailed information on the age and sex distribution is presented in Table 1.

**Table 1:** Samples distribution by sex and age

Age	Boys	Girls	Total
6	227	206	433
7	380	332	712
8	353	263	616
9	257	280	537
10	232	279	511
11	267	411	678
12	413	512	925
13	344	616	960
14	257	406	663
15	273	374	647
16	177	290	467
17	198	302	500
18	247	402	649
19	227	471	698
20	202	388	590
Total	4054	5532	9586

### Model architects and parameters

In the present experiments, we selected the VGG network VGG16 to perform the image classification tasks. The residual network (ResNet) has deeper network layers than the traditional convolutional networks; therefore, armed with moderate depths, ResNet101 was also employed to fulfill the current work.

### Dataset’s pre-treatment

Based on the existing dataset, it is necessary to preprocess each dental image to better substitute it into the model for deep learning. In the first step, we extracted the key parameters of each OPG according to sex and age, and concurrently, we removed related OPGs missing any of the key parameters, as this will affect the training effect of the model. We selected the EasyOCR image recognition engine to extract words and divide a valid dataset into different groups according to age and sex. It contains several steps, including image preprocessing (denoising, color saturation, and sharpness fixing), CRAFT text detection, intermediate processing (such as tilt processing), text recognition, post-processing, and output of the result. Detailed information is available online (https://github.com/JaidedAI/EasyOCR, version 1.4.1).

After classification, we used the Open CV (OpenCV-python 4.5.4.60) tool to bilinearly interpolate images to 500 × 500 grayscale plots and 224 × 224 three-channel pictures and stored them separately for the subsequent ResNet101 and VGG tasks.

In the following VGG and ResNet experiments, we used 10% of each category as the validation set, 20% as the test set, and the remaining 70% as the training set.

### VGG16 setting

**Fig. 1:**
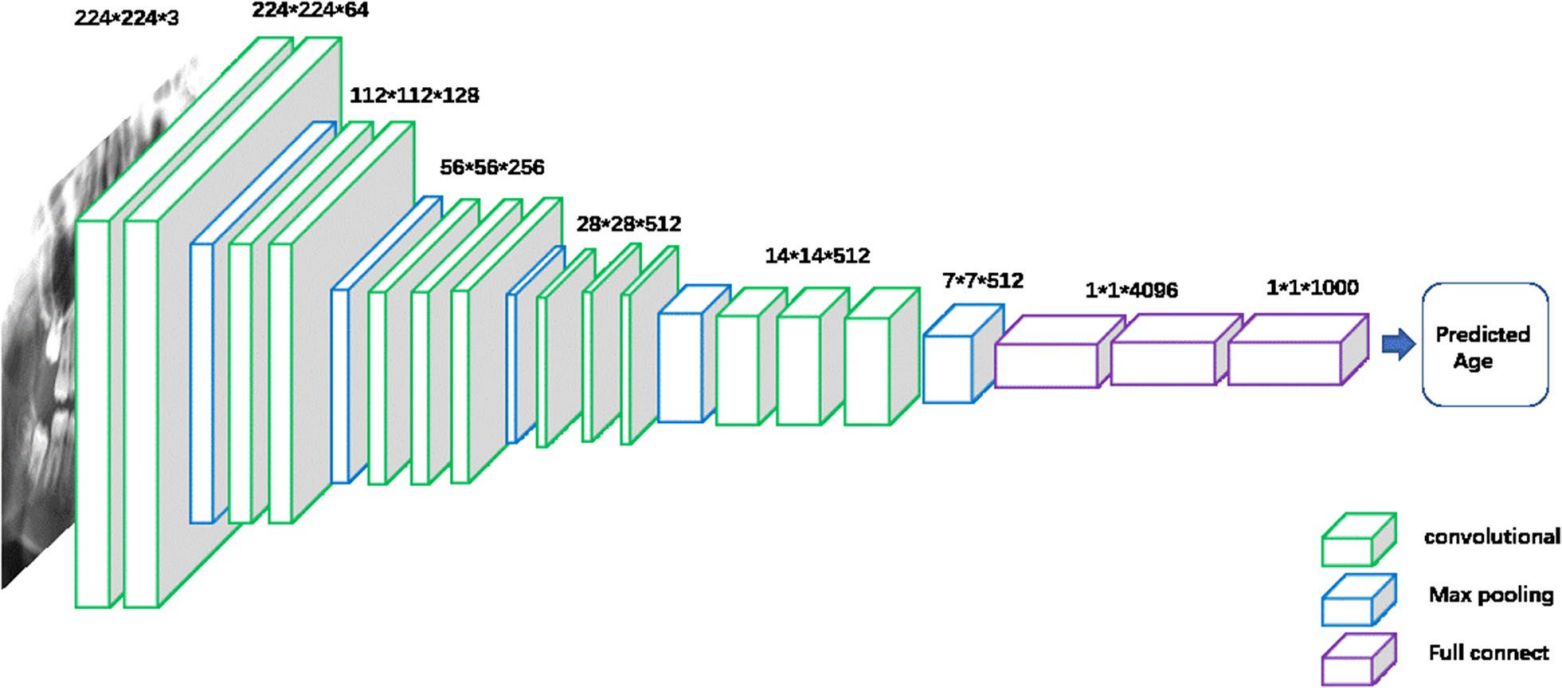
The basic structure of VGG16 network

The VGG16 model is illustrated in Fig. 1. The detailed parameters of the kernel structure and the output of the corresponding layers are listed in Additional file 1: Table S1. The VGG16 model consists of five layers. In the first two layers, each layer contained two convolutional layers, and in the rest three layers, each layer had three convolutional layers. It consists of three convolutional layers with 13-parameter update layers. Each layer ends with a max-pooling layer for feature extraction. The five layers were output with 64, 128, 256, 512, and 512 channels respectively, and a 512 × 7 × 7 channel output was obtained, which was passed into the three-layer fully connected layer to obtain the output classification. The VGG16 model consists of 16-parameter update layers, where the output dimension of the last fully connected layer can be specified. In the present study, the 6–20-year-old dataset was divided into 15 categories at a 1-year interval for multi-class training.

Adopting the VGG16 network required additional processing of the dataset. Based on the original OPG images, each image was scaled to 224 × 224 size by bilinear interpolation, which is described in the dataset’s pre-treatment section. Simultaneously, the pictures maintain the RGB three-channel mode input network. Approximately 70% of the overall data set was used as the training image set for model training and parameter update, and 20% was used as the test image set for iterative optimization of model parameters during the training process, and the remaining part of the image set was used to verify the final model training effect. The model training was updated using an optimizer with a learning rate of 1e-5, a batch size of 1, and a total of 50 iterations.

### ResNet101 setting

**Fig. 2:**
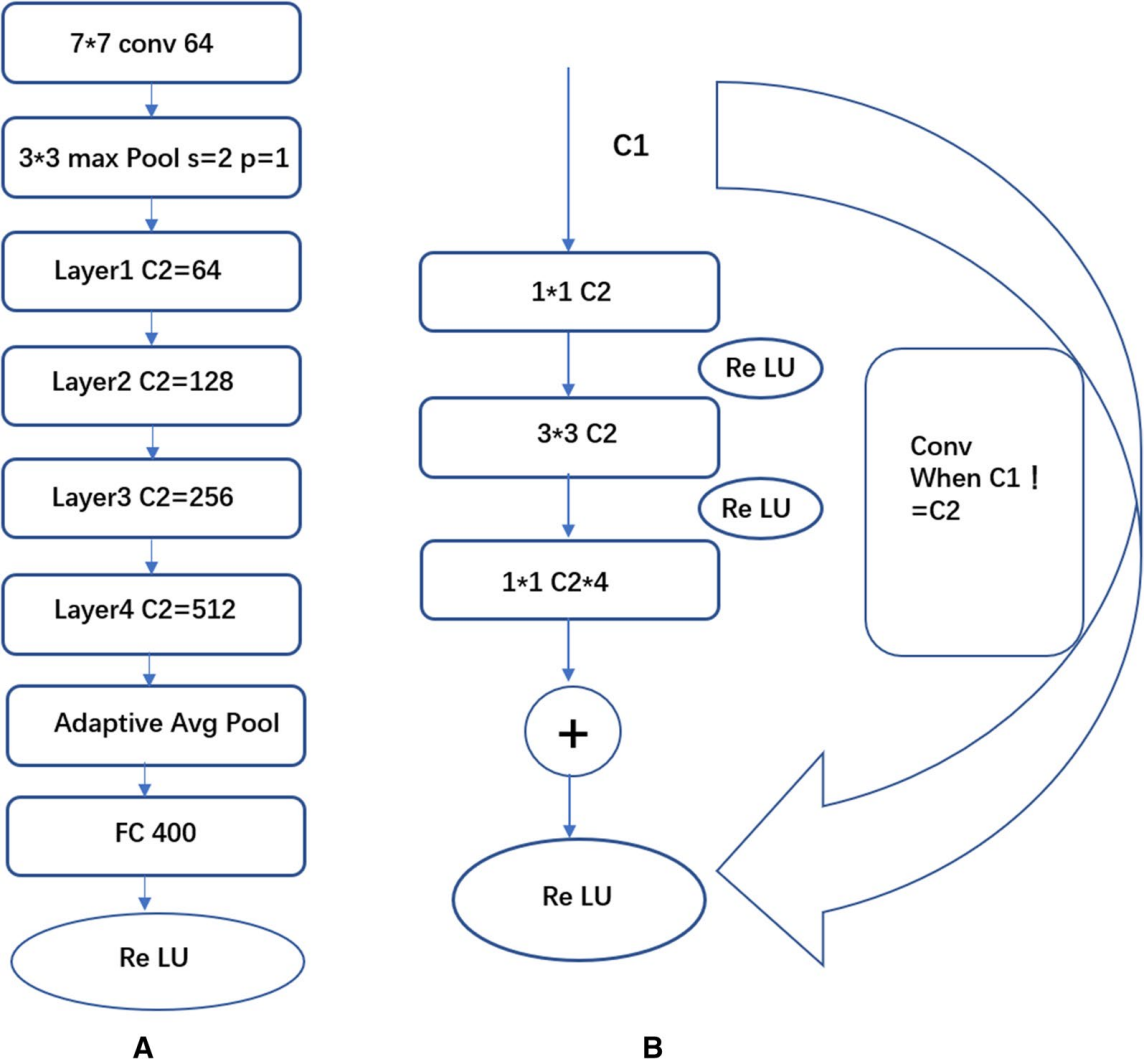
ResNet101 network structure diagram (**A**) and its BottleNeck unit (**B**)

The ResNet101 network consists of 101-parameter update layers, as shown in Fig. 2. The key is the four residual blocks in the middle, which are represented as layer 1, layer 2, layer 3, and layer 4. Each of these layers is a ResNet generic bottleneck, as shown in Fig. 2 (right). In bottleneck, the number of input channels is C1, which is first reduced to C2 through a 1 × 1 convolution kernel, then a 3 × 3 convolution kernel for the convolution operation, and finally a 1 × 1 convolution kernel to increase the dimension to four times that of the C2 channel. Simultaneously, bottleneck determined the number of channels C1 and C2. If they are not equal, the input of C1 will be converted to the input of C2 as the residual after a round of convolution operation. Otherwise, C1 is directly mapped to the residual, and the residual will be learning is done by adding to the bottleneck’s own output. Several such bottlenecks were experienced in each layer. In ResNet101, the C2 parameters selected for the four layers were 64, 128, 256, and 512. After all residual blocks are updated, the network is fully connected to the 400-channel output after one round of average pooling. Considering the sex differences in tooth age and the increase in the model prediction accuracy, based on the original ResNet101, sex parameters were added for training. After merging the sex and network output results, a full connection operation was performed, and the new model structure is shown in Additional file 1: Fig. S1.

The initial learning rate of the optimizer for the ResNet101 network was set to 0.001 and the momentum was set to 0.9. The network scheduler was set to step size 12, and the gamma value was set to 0.5 to dynamically adjust the learning rate during the training process to achieve a better training effect. These numbers represent default parameters for regular machine learning. In terms of network training, the number of iterative trainings is 30, and the batch input size is four; thus, the input size of the input network is actually 4 × 1 × 500 × 500, and the sex parameter also needs to be converted to a 4 × 1 matrix input network.

### Model training loss calculation and tuning

The loss function calculation of the VGG16 model adopts a cross-entropy loss calculation function, which is suitable for multi-classification problems. The calculation formula is as follows:

1 $$Loss\left( {x,class} \right) = - ln\frac{{\exp \left( {x\left[ {class} \right]} \right)}}{{\mathop \sum \nolimits_{i = 0}^{14} \exp \left( {x\left[ j \right]} \right)}}$$

In Eq. (1), class is the index of the predicted classification of the current sample in the overall classification table, and x[j] represents the predicted j-th output.

The loss function calculation of the modified version of the ResNet101 model adopted the variance loss calculation function, and the calculation formula was as follows:

2 $$batchLoss\left( i \right) = \mathop \sum \limits_{i = 0}^{batchSize - 1} \left( {output\left[ i \right] - y\left[ i \right]} \right)2$$

3 $$totalLoss = \frac{{\mathop \sum \nolimits_{i = 0}^{batchNum - 1} batchLoss\left( i \right)}}{N}$$

In Eqs. (2) and (3), batchLoss is the loss of a batch training result, batchSize is the batch size (4), output[i] is the output result of the sample model with index i in the batch, and y[i] is the actual y value of the sample. totalLoss is the total loss for one iteration, batchNum is the total number of batches, and N is the total number of training set samples.

In CNNs, the loss function is used to calculate the deviation of the CNN output from the label result, which is then used in the backpropagation process to update the gradient. By continuously training and optimizing the parameters in the CNN, the goal is to minimize the loss function and finally obtain the best CNN model. This is an important parameter for evaluating the efficacy of the current model. Normally, the larger the loss function value, the worse the result of the estimate.

During the model training process, we attempted to find the best model with the smallest possible loss, perform multiple iterations by adjusting the epoch size, and find the best iterative model. Simultaneously, the appropriate optimization parameters were selected in the model parameter update optimizer to dynamically adjust the learning rate to achieve the best effect.

The experimental environment used in this study was a personal computer. Additional file 1: Table S2 lists the software and hardware environments used in this experiment.

**Table 2:** Comparison of the effect reference value of two network dental age prediction models

Age range	Network model	Precision (%)	Recall (%)	Accuracy (%)	F1-Score (%)
6–8	ResNet101-gender	80.32	53.84	88.73	64.47
	VGG16	83.42	82.89	93.63	83.19
9–11	ResNet101-gender	43.57	46.42	80.06	44.95
	VGG16	61.07	60.71	86.32	60.89
12–14	ResNet101-gender	53.03	28.68	75.36	37.23
	VGG16	63	63.52	81.21	63.26
15–17	ResNet101-gender	31.53	48.52	72.33	38.22
	VGG16	45.54	54.43	80.48	49.59
18–20	ResNet101-gender	54.71	74.35	82.21	63.04
	VGG16	71.6	59.48	86.95	64.98

Three parameters of VGG16 and ResNet101 have been described in Table 2. Recall, accuracy and precision equations have been listed as follows:

$$Recall = \frac{TP}{{TP + FN}}$$

$$Accuray = \frac{TP + TN}{{TP + FN + FP + FN}}$$

$$Precision = \frac{TP}{{TP + FP}}$$

$$F1 - score = 2/\left( {\frac{1}{precison} + \frac{1}{recall}} \right)$$

By calculating recall and precision, we obtained the F1 score of the model in the last step. The precision, recall, accuracy and F1-score of the two models were compared and tested to obtain a better CNN for age estimation. An age threshold was selected to assess the two models. When the actual estimated age was generated by the CNN model, the age difference between chronological ages was calculated, and the absolute error was counted by an interval of one year. The detailed information is listed in Table 3.

**Table 3:** Age threshold of the two CNN models

Age threshold	Accuracy	
	VGG16	ResNet101
< 1	0.48	0.31
< 2	0.79	0.61
< 3	0.91	0.79
< 4	0.95	0.88
< 5	0.97	0.95
< 6	0.99	0.98
< 7	0.99	0.99
< 8	0.99	0.99
< 9	1	1

## Results

### VGG16 network

First, the VGG network was trained for 50 epochs and the accuracy of the model was validated after each epoch. The accuracy of the prediction rate of the model is shown in Additional file 1: Fig. S2. Among them, we selected the 40th epoch (the index on the way was 39) to train the model for subsequent experiments, and the accuracy rate was 0.382.

Next, we selected the model corresponding to the maximum value of the correct rate for the experiments and set the allowable error range at different thresholds. The VGG16 network was better for multi-classification problems, and the actual error was between 1 and 1.99 with an accuracy rate of 79%.

The results of VGG16 within the five age thresholds (6–8, 9–11, 12–14, 15–17, and 18–20) in the samples were shown in Table 2. The VGG16 network had the highest prediction accuracy in the age range of 6–8 at 93.63%, and the model F1 score was also the highest. The accuracy of age threshold < 3 years old is 91%. Age threshold analysis implies that the greatest age difference is between 2 and 3 years.

### ResNet101

We trained the ResNet101 network for 30 epochs and recorded the model validation variance loss after each epoch. The loss curve for the model is shown in Additional file 1: Fig. S3. Among them, the 30th epoch (index 30 in the figure) had the lowest variance loss of 0.0136.

To obtain the accuracy rates under different error thresholds, we selected the model corresponding to the minimum loss value for experiments and checked the validation set. The ResNet101 network has an accuracy rate of 61% between 1 and 1.99 years old, which is slightly lower than the VGG16 network.

We also calculated the performance of the ResNet101 network in terms of the recall, accuracy, precision, and F1 score, as shown in Table 2. The ResNet101 network also exhibited the highest accuracy and F1 score in the 6–8 age range.

In general, in terms of the accuracy of various age ranges and F1 scores, the prediction results of the VGG16 network showed a better performance than those of the ResNet101 network. However, in the 15–17-year-old group, the model effect of VGG16 was worse than other ranges, specifically with an F1 score of only 49.59, while the rest was above 60%. For younger age groups, the model prediction results of the VGG16 network were relatively good. The accuracy of the VGG16 model can reach 93.63% in the 6–8-year-old group, which was better than the 88.726% of the ResNet101 network. The accuracy of the age threshold < 4 years was 88%. Age threshold analysis indicated that the greatest age difference was between 3 and 4 years.

## Discussion

Age estimation plays a key role in aiding arbitrament when encountering circumstances such as civil or criminal affairs, especially in teenagers’ social events [13, 21, 22]. Therefore, there is an urgent demand for a fast and accurate method or model for age estimation. The Demirjian or Willems method, as representatives of traditional approaches, still has some limitations, such as lacking the region’s special modifications to achieve satisfaction and losing its power in larger groups, such as in > 16 years old. Moreover, traditional manual methods are usually labor-intensive and time-consuming for daily clinical activities [16, 21, 23]. Overcoming and addressing these issues are challenging for colleagues in the DA estimation community.

CNN have shown strong competitive potential for handling complicated medical images over the past few years [24–26]. The CNN uses a convolution operation as the core and integrates data points into a fixed receptive field, thereby retaining any local information in the data. Simultaneously, convolution operations and pooling operations greatly reduce the complexity of the original data and, to a certain extent, reduce the number of parameters inside the network. Therefore, it is ideal to extract the characteristics of the tested tooth in this study for tasks such as identification or detection, as mentioned in the Introduction section.

As special network architectures of CNN, the Visual Geometry Group (VGG) and ResNet show excellent performance in picture classification-related problems. They are used to extract some medical features from the picture and output them as feature vectors. It is suitable for extracting the characteristics of several medical images for identification or detection. The outstanding branches of CNN, VGG16, and ReNet101 are equipped with special network architectures in general convolution neural networks. Armed with the ResNet network, the famous AlphaGo AI model defeated the famous world chess champion. Hence, we employed these to demonstrate the VGG16 and ResNet101 networks in the current study.

Examining DA estimation involving deep learning, some scholars have inspired us to work on the current paper. To the best of our knowledge, besides the abovementioned advances in the Introduction section, few other impressive studies have been reported online. Guo YC et al. conducted a thorough investigation of 10,257 samples ranging from 5 to 24 years old [23]. Compared with the manual method, employed machine learning methods such as EfficientNet-B1, EfficientNet-B3, EfficientNet-B5, and Se-ResNet101 had a more accurate outcome when performing age classification without human interference. Wu TJ et al. applied AI-assisted standards to gain a more accurate chronological age prediction with mean errors of < 0.05 years in children with growth delay [27]. Moreover, to further improve the accuracy, Sharifonnasabi F et al. proposed a method of image classifiers and a hybrid model based on CNNs and K-nearest neighbors (KNN); their innovative model (HCNN-KNN) managed to obtain high accuracies with satisfaction [28]. Wang et al. developed DENSEN based on a Soft Stagewise Regression Network for both juveniles and older adults [29]. In their studies of the 3–11 (children), 12–18 (teens), 19–25 (young adults), and 25 + (adults) groups, DENSEN produced MAEs of 0.6885, 0.7615, 1.3502, and 2.8770, respectively. DENSEN proved to have less laboratory time, and it is an open-source model that can be widely adopted. In addition to age estimation, CNN-assisted radio-diagnostic use also shows discriminant power to distinguish males and females with excellent accuracy, according to Ademir et al.’s work [30]. Furthermore, a Malaysian study used an artificial neural network multi-layer perceptron (ANN-MLP) based on a population-specific prediction model, and ideal age prediction results were obtained. It has potential applications in DA estimation [31].

In China, important legal age thresholds (14, 16, and 18 years) are lines of demarcation for civil, legal, and criminal scenarios. Traditional DA evaluation methods rely on time-consuming manual processes that can be affected by subjectivity. For this reason, we skipped the manual evaluation phase to introduce CNNs with OPGs directly for a collaboration between two different disciplines. In the current study, we employed two different CNN models, VGG16 and ResNet101, to investigate their power in the eastern population of China. To the best of our knowledge, the VGG network was the first to test its performance in the community of DA estimation. Compared with ResNet101, VGG16 outperformed ResNet101 in all groups. Considering that another study by Guo YC et al. in a northern population of China [23] had a high accuracy of > 90%, it is clear that certain factors may have led to an inferior performance compared with the results of Guo YC et al.’ study: First, detailed selected model differences may be incurred which will be reflected in the outcomes. In their classification performance of the logistic regression models to determine an individual’s age, one was younger than the legal age threshold (Y = 0) or equal to the legal age threshold or older (Y = 1). Binary judgment design may help improve accuracy and avoid unnecessary interference. Second, we had more age groups, while they only focused on three important age thresholds (14, 16, and 18 years). Different age classifications may also lead to higher accuracy according to the data provided. The two CNN models that we selected were easy to construct and did not require complicated hard and soft environments. Compared with traditional manual evaluation methods, our methods are labor-efficient, and a CNN-related age estimation website based on these two methods will be developed in the future to improve society.

It is possible to train the feature map of the convolutional network by extracting the convolutional layer results. Thus, the feature extraction effect was employed to obtain a comprehensive glimpse of the CNN model. The training feature map after a convolutional layer of ResNet101 is shown in Additional file 1: Fig. S4; by this feature map, we found that convolutional networks have obvious advantages in the extraction of peripheral tooth features, as the highlighted parts of the feature map are concentrated in the crown, tooth root, and surrounding parts and ignore similar parts between samples, while the neural network can consider some features that will not be considered by manual calculation, such as the nasal bone.

Instead of the manual label in the region of interest of OPGs, the two CNN models directly showed their potential as useful tools for DA investigation. It is a great leap compared with traditional manual age estimation methods because it saves time and manpower resources. Our study showed that the detailed connection and interpretation of AI-guided features would be of great interest to the DA assessment community. Moreover, when an expected CNN model, such as a VGG, is achieved, a region-suitable teenager age estimation method will help dentists evaluate tooth development and decide subsequent treatments.

Although the current study has proven the potential of CNNs in assessing the chronological age of teenagers via OPGs, it still has certain limitations. First, we have not used targeted segmentation techniques to optimize and attempt machine-guided learning to evaluate chronological age. As automated image segmentation manages to obtain better predictions, we will investigate the reliability of DA assessments in future studies. Second, this was a descriptive study conducted in an eastern population of China, and performances may vary while applying it to other ethnic populations despite the same CNN models. Therefore, further clinical and prospective studies in different countries or regions are required. Third, according to the corresponding curve, 40 and 30 epochs achieved almost the best accuracy and loss; thus, these epochs are taken as the final index. Admittedly, adding extra epochs, such as 100 epochs, may provide a broad and deep understanding.

## Conclusions

In conclusion, compared with the ResNet101 network, VGG16 showed better performance in DA estimation via OPGs from a whole scale. CNNs such as VGG16 hold great promise in forensic science and clinical practice.

## Supplementary Information

**Additional file 1**. Supplementary tables and figures.

## Data Availability

The datasets used and/or analyzed during the current study available from the corresponding author on reasonable request.
