# Supplementary material for: A population-based study to assess two convolutional neural networks for dental age estimation
Source: BMC Oral Health. 2023 Feb 17;23:109. doi: 10.1186/s12903-023-02817-2 (PMC9938587; doi:10.1186/s12903-023-02817-2)
Supplement: Supplementary file 1 — Additional file 1. Supplementary tables and figures. [file 12903_2023_2817_MOESM1_ESM.docx]

Table S1 Parameters of layers、kernel structure and output of VGG16

| Layer | Kernel Structure | Output |
| --- | --- | --- |
| Input |  | 224*224*3 |
| Convolutional | 3*3 | 224*224*64 |
| Convolutional | 3*3 | 224*224*64 |
| Max-Pool | 2*2 | 112*112*64 |
| Convolutional | 3*3 | 112*112*128 |
| Convolutional | 3*3 | 112*112*128 |
| Max-Pool | 2*2 | 56*56*128 |
| Convolutional | 3*3 | 56*56*256 |
| Convolutional | 3*3 | 56*56*256 |
| Convolutional | 3*3 | 56*56*256 |
| Max-Pool | 2*2 | 28*28*256 |
| Convolutional | 3*3 | 28*28*512 |
| Convolutional | 3*3 | 28*28*512 |
| Convolutional | 3*3 | 28*28*512 |
| Max-Pool | 2*2 | 14*14*512 |
| Convolutional | 3*3 | 14*14*512 |
| Convolutional | 3*3 | 14*14*512 |
| Convolutional | 3*3 | 14*14*512 |
| Max-Pool | 2*2 | 7*7*512 |
| Full Connect |  | 4096 |
| Full Connect |  | 4096 |
| Full Connect |  | 1000 |

|  | Table S2 Experimental environment | |  |
| --- | --- | --- | --- |
| Type | Name | Version | Information |
| Software | Pytorch | 1.10.1 | Open-source |
|  | Numpy | 1.21.4 | Open-source |
|  | OpenCV-Python | 4.5.4.60 | Open-source |
|  | Python | 3.8 | Open-source |
| Hardware and Drive | CPU | Core i7-10875H | INTEL, USA |
|  | GPU | GeForce RTX 2060 | NVIDIA, USA |
|  | CUDA | NVIDIA CUDA 11.4.141 | NVIDIA, USA |


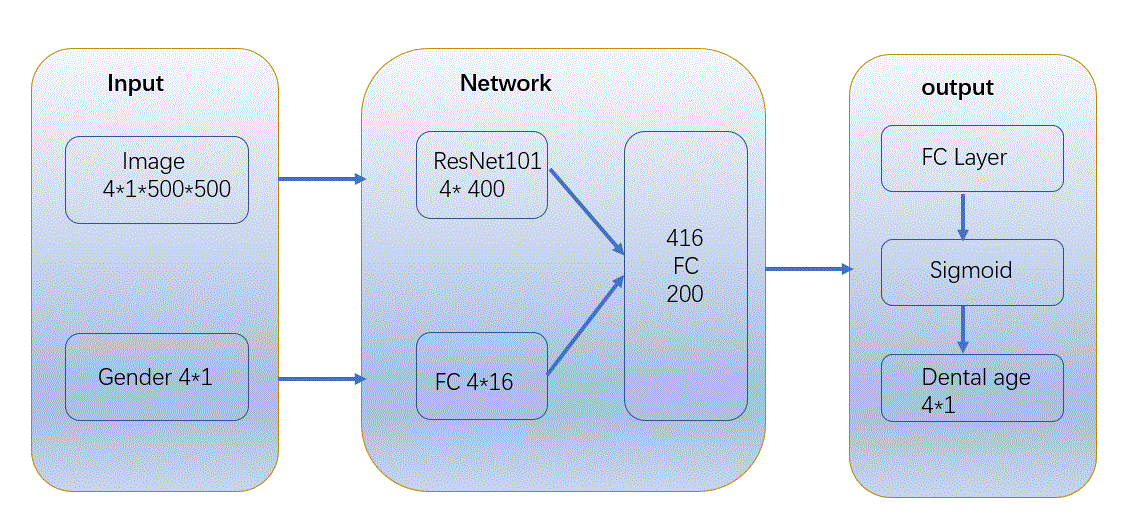


Fig.S1 ResNet101 network’s structure with gender parameters’ design


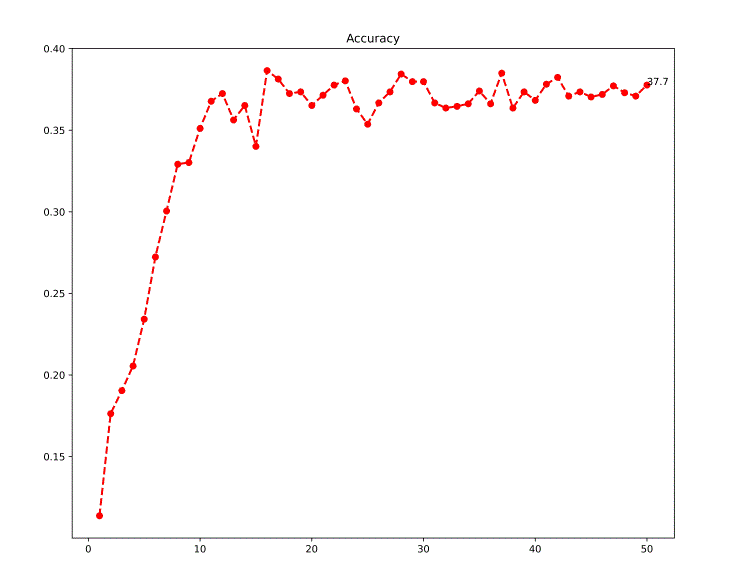


Fig.S2 VGG network training accuracy results


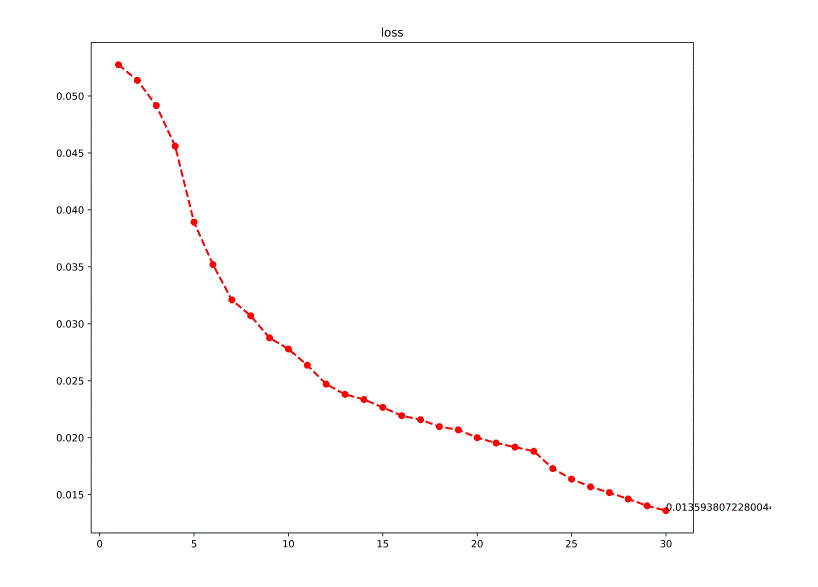


Fig.S3 ResNet network training Loss curve results


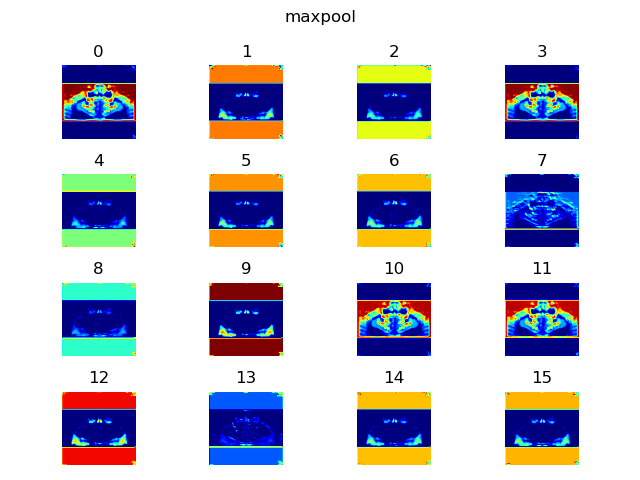


Fig.S4 Features of convolutional network training
